# A Dose–Response Study on the Relationship between White Meat Intake and Metabolic Dysfunction-Associated Steatotic Liver Disease (MASLD) in Southern Italy: Results from the Nutrihep Study

**DOI:** 10.3390/nu16183094

**Published:** 2024-09-13

**Authors:** Davide Guido, Nicole Cerabino, Martina Di Chito, Rossella Donghia, Cristiana Randazzo, Caterina Bonfiglio, Gianluigi Giannelli, Giovanni De Pergola

**Affiliations:** 1 Unit of Data Science, National Institute of Gastroenterology “Saverio de Bellis”, IRCCS Hospital, Castellana Grotte, 70013 Bari, Italy; rossella.donghia@irccsdebellis.it (R.D.); catia.bonfiglio@irccsdebellis.it (C.B.); 2 Center of Nutrition for the Research and the Care of Obesity and Metabolic Diseases, National Institute of Gastroenterology IRCCS “Saverio de Bellis”, Castellana Grotte, 70013 Bari, Italy; giovanni.depergola@irccsdebellis.it; 3 Dipartimento di Promozione della Salute, Materno-Infantile, Medicina Interna e Specialistica di Eccellenza (PROMISE), University of Palermo, I-90127 Palermo, Italy; cristiana.randazzo@unipa.it; 4 Unit of Clinical Nutrition, Obesity and Metabolic Diseases, University Hospital Policlinico “P. Giaccone”, I-90127 Palermo, Italy; 5 Scientific Direction, National Institute of Gastroenterology “Saverio de Bellis”, IRCCS Hospital, Castellana Grotte, 70013 Bari, Italy; gianluigi.giannelli@irccsdebellis.it

**Keywords:** MASLD, white meat intake, survey, dose–response modeling, logistic regression, DAG

## Abstract

(1) Background: Metabolic dysfunction-associated liver disease (MASLD) is one of the most important causes of liver disease worldwide. Meat consumption is a growing trend and white meat has been shown to have beneficial effects on cardiometabolic risk factors. The aim of this study was to investigate the dose–response relationship between white meat intake and MASLD at survey level in a Southern Italy setting. (2) Methods: This cross-sectional study encompassed 1192 subjects (509 males, 42.7%) without missing data from the second wave of the NUTRIHEP cohort (2014–2016). Adjusted dose–response modeling was employed for statistical analysis; (3) Results: There were 587 subjects with MASLD (49.2%), i.e., 278 males (54.6%) and 309 females (45.2%). By increasing the intake, an unfavorable influence of white meat on MASLD was significantly revealed in females, whereas a protective effect of white meat was detectable in males. Male sex was shown to be involved in other associations in this study, such as influencing the preference for specific foods such as poultry and chicken skin. (4) Conclusions: Our data suggest that white meat does not have a clear-cut independent dose–response effect on MASLD, but sex may be a trigger moderator for age and BMI, with an increasing unfavorable effect of white meat in women, and a favorable effect in men.

## 1. Introduction

Metabolic dysfunction-associated liver disease (MASLD), previously known as nonalcoholic fatty liver disease (NAFLD) [1,2] is a condition characterized and defined by the accumulation of lipids within hepatocytes in an amount that affects at least 5% of the liver [3]. However, the definition of MASLD is “negative” because in order to diagnose it, other causes of chronic liver disease such as hepatitis B and C virus infections must be excluded, as well as significant and risky alcohol consumption [4].

MASLD is one of the most important causes of liver disease worldwide and is likely to emerge as the leading cause of end-stage liver disease. Over 30% of people worldwide suffer from MASLD, and the incidence is as high as 70% in people with metabolic disorders [5,6]. In fact, the epidemiology and demographic characteristics of MASLD vary worldwide, usually in parallel with the prevalence of obesity, DMT2 and cardiovascular diseases (CVD) [5,6].

A diet rich in calories, inactivity, and insulin resistance (IR) are the primary triggers of MASLD, as well as background genetic factors and exposure to environmental risk factors and [5,6].

Meat consumption has been part of the human diet for over 2.5 million years, but there is a growing trend towards vegetarian or vegan diets due to the health benefits of plant-based diets. Meat contains essential amino acids, complete protein and high amounts of micronutrients such as vitamin B12, iron, zinc, selenium, iodine and vitamin D. It also contains essential fatty acids, especially in the lean parts [7].

As a part of meat consumption, unprocessed, fresh, lean white meat has been shown to have beneficial effects on cardiometabolic risk factors [8,9]. In particular, consuming mostly white meat was inversely associated with incident CVD [9].

It should be noted that the ATTICA study showed that participants who mostly consumed white meat had increased adherence to the Mediterranean diet [9]. Furthermore, white meat (and poultry in particular) is considered moderately protective or neutral with respect to cancer risk [7]. White meat consumption has mainly been inversely associated with the incidence of gastric cancer [10] and hepatocellular carcinoma [11,12].

It is noteworthy that there is no definition of white meat in the WCRF/AICR report, and “white meat” is commonly intended to mean poultry (chicken, turkey, duck and goose) and rabbit in scientific studies [13].

Nutrition is a modifiable risk factor that plays an important role in preventing or delaying the onset of MASLD. However, whether or not high consumption of red and/or processed meat is associated with a higher risk of MASLD [14], no significant association between white meat and MASLD has been shown [14]. In addition, metabolic dysfunction-associated steatohepatitis (MASH) is an unfavorable inflammatory evolution of MASLD and, with regard to food consumption, the MASH pattern compared with NO-MASH pattern was characterized by a lower intake of white meat in a large population of people with type 2 diabetes [15].

Interestingly, no dose–response studies have been conducted to examine the associations between white meat consumption and MASLD by exploring the potential ranges and conditions that could make exposure a risk or protective factor. Given this background, the aim of this study was to investigate the dose–response relationship between white meat intake and MASLD at survey level in a Southern Italy setting, by adjusting for a number of selected confounders. Secondly, associations with specific types of white meat were also evaluated.

In synthesis, the rationale for examining specifically white meat was (1) to gain more information about the possible influence of white meat consumption on the presence of MASLD and (2) to know whether a dose–response effect exists concerning the possible impact of white meat on the development MASLD.

## 2. Materials and Methods

### 2.1. Study Population and Study Design

The NUTRIHEP study is a survey carried out by extracting the medical records of general practitioners in the municipality of Putignano (subjects ≥ 18 years) to minimize errors regarding the distribution of the population sample by age and gender. It should be noted that Italian law requires the presence of a family doctor, allowing for the overlap of medical and census data. Participants were interviewed by trained physicians and/or nutritionists to collect information on their sociodemographic characteristics, health status, personal history, and lifestyle factors such as smoking, education level (International Standard Classification of Education), marital status, and eating habits [16]. There were two NUTRIHEP waves: the first was applied at baseline in 2004–2005, and the second was obtained from follow up measurements from 2014 to 2018, in which all eligible subjects of the first wave were invited to take part. The subjects underwent the same protocol as at the first enrollment.

The methodological details of this population-based study have been described in a previously published paper [17]. The study was conducted in accordance with the Helsinki Declaration of 1975. The manuscript was organized according to the “Strengthening the Reporting of Observational Studies in Epidemiology–Nutritional Epidemiology” (STROBE-nut) guidelines [18], and participants signed informed consent forms before undergoing examination.

All participants signed informed consent forms after receiving complete information about their medical data to be studied. Those enrolled were included in this analysis, investigating a total of 1426 subjects. The study was approved by the Ethical Committee of the Minister of Health (DDG-CE 502/2005; DDG-CE-792/2014, on 14 February 2014) [17].

The study design was cross-sectional considering only the second wave.

### 2.2. Dietary Assessment, Exposure and Outcome

The validated European Prospective Investigation into Cancer and Nutrition (EPIC) food frequency questionnaire was administered during the visit, and each food item (260 food items) was converted into an average daily intake in grams, as previously carried out for other studies [17]. Individual nutrient intakes were derived from foods included in the dietary questionnaires through the standardized EPIC Nutrient database [19].

White meat intake was computed by summing the intake (g/die) of the relative food group, items including white stewed meat, roast white meat, white cutlet, white meat slices, rare white meat steak, medium white meat steak, well done white meat steak, white meat hamburger, white meat meatballs, chicken thigh, chicken breast, chicken—other parts, poultry, chicken skin, and rabbit. Notably, we defined white meat as poultry, chicken, duck, turkey and rabbit on the basis of the definition of white meat provided by previous studies [13].

MASLD was diagnosed in subjects with hepatic steatosis on ultrasound, without AFL (alcoholic fatty liver; the cutoffs for AFL were 30 g/day for men and 20 g/day for women) [5,17]; drug-induced fatty liver disease (e.g., corticosteroids, valproic acid, amiodarone); hepatitis C or B viral infections; or other disorders [20].

### 2.3. Potential Confounders

#### 2.3.1. Food Groups and Single Item Foods

Food groups were formed by summarizing the daily intake of single foods. A number of composite indicators were created [21] such as legumes, vegetables, dairy foods, red meat, processed meat, fish and seafood, fruits, fried foods, grains, soft drinks, and sugar foods. All food groups with relative items are presented in Table S1. In addition to food groups, single item foods such as eggs (g/die), margarine (g/die), alcohol, (g/die) were also considered in the set of potential confounders.

#### 2.3.2. Other Potential Confounders

In addition to the food confounders, a number of other variables were considered as potential confounders: age, sex, education (high school degree: yes/no), body mass index (BMI), smoking habit (0 = no, 1 = yes), diabetes, total cholesterol, and daily kcal intake.

### 2.4. Statistical Analysis

Subjects’ characteristics are reported as mean and standard deviation (mean ± SD) for continuous variables, and as frequency and percentage (%) for categorical variables. Descriptive statistics based on the quartiles of white meat intake (g/die) were also computed overall and stratified for sex, in order to preliminarily assess the dose trend.

Firstly, raw dose–response modeling was applied on MASLD (0 = no, 1 = yes) by considering the white meat intake categorized in quartiles, overall and stratified by sex. In this case, simple and confounder-adjusted models were fitted. The set of confounders included in the modeling was determined by a DAG-based causal inference procedure [22,23,24]. In detail, confounding was mitigated by using a theory-driven methodology that provided a minimal confounder adjusting set, to be included in the dose–response modeling. Potential confounders were preliminarily identified by the literature in relation to studies performed in similar settings [25,26].

Hence, to determine the shape and magnitude of the relationship between white meat intake (g/die) and MASLD, adjusted statistical models were fitted. In detail, dose–response modeling for dichotomous outcomes were fitted [27,28]. Missing values were managed by complete case analysis (i.e., listwise deletion). In order to evaluate the above-specified relationship, the white meat intake was smoothed by a cubic spline function, by using 9 knots on the percentiles 0% (or 1%), 12.5%, 25%, 37.5%, 50%, 62.5%, 75%, 87.5%, and 100% (or 99%) [29,30]. The reference value of the white meat regressor was the median. In this case, the odds ratio (OR) of the dose–response relationships (with 95% confidence bounds, i.e., confidence intervals) were plotted in continuous shape in relation to white meat intake (g/die), and the evaluation in relation to OR = 1 was considered for the significance. The adjustment covariates were fixed to the median values, mode, and reference category in relation to their continuous, categorical and dichotomous nature [31]. In addition, to confirm the output, generalized additive models (GAM) [30] were also fitted and parametrized on the binomial distribution returning OR as the effect size.

Of note, in order to better evaluate the dose–response relationship, associations between confounders and exposure factor were investigated. In detail, correlation coefficients, mean differences (MD) and OR were tested, as appropriate. Also, in order to check multicollinearity, Variance Inflation Factor (VIF) was evaluated, and confounders with VIF > 5 were discarded.

Then, interaction dose–response effects were also evaluated by probing sex (1 = M), age (y) and BMI (kg/m^2^) [32] as moderators. In particular, the plot regarding this step of the analysis involved the OR of MASLD in relation to white meat intake, by varying the moderator. Notably, concerning the sex variable, due to its dichotomous nature, the plot was reversed, by representing the sex effect in relation to white meat intake. This did not change the interpretation of the modification effect given the statistical modeling equation, in which the interaction term is the product of the main effects.

Finally, an in-depth analysis was performed by linking white meat consumption with the “grains” food group (g/die) (as a proxy of carbohydrates): in detail, at first subjects were stratified on the quartiles of white meat intake and descriptive (mean ± SD) and inferential (as one-way ANOVA to compare the strata) statistics were computed for each “grains” food. Secondly, interaction dose–response models on MASLD (on the whole sample and by sex) were fitted by considering the “grains” food group (g/die) [32] as moderators. Statistical significance was set at *p*-value < 0.05. *p*-values between 0.05 and 0.10 were also reported as suggestive. All analyses were performed using R software (version 4.3.3) [31], and its packages dagitty [32], rms [33], mgcv [34], and interactionRCS [35].

## 3. Results

At first, a listwise deletion was applied to manage the missing data by retaining n = 1192 subjects (509 males, 42.7%) in the analysis. The number of subjects with MASLD was 587 (49.2%), i.e., 278 males (54.6%) and 309 females (45.2%). Table 1 shows the descriptive statistics of the exposure-related items, i.e., white meat compound intake with its elements, MASLD and potential confounders, in terms of mean ± SD or frequencies, as appropriate. Notably, all white meat single foods reported large SD values and coefficients of variation bigger than 1 (overdispersion), because they were zero-inflated.

It shows some significant differences in the consumption of some kind of white meat between men and women (Table 2). In particular, men overall eat a higher amount of poultry, chicken skin and rabbit, and a lower amount of meat meatballs and white meat hamburger. These findings confirm that the preference for specific foods may be influenced by sex. In Table S2 the pairwise association between exposure and confounders is shown.

Remarkably, in males a significant protective effect of white meat was revealed (OR = 0.985, *p* = 0.005) on both linear effect and in the higher exposure categories (OR_q3_ = 0.502, *p* = 0.041; OR_q4_ = 0.319, *p* = 0.002). In contrast, in females the trend favored MASLD, until a suggestive OR_q4_ was equal to 1.780 (*p* = 0.067) (Table 3).

Concerning single food items, white meat slice intake had a protective effect OR = 0.896 in males. Concerning females, roast white meat intake presented a linear OR equal to 1.036 (*p* = 0.067).

Notably, for the categorization of single food items in percentiles, because of the sparsity, a threshold was applied only in relation to the highest first non-zero percentile by jointly considering overall and sex-stratified samples.

**Figure 1:**
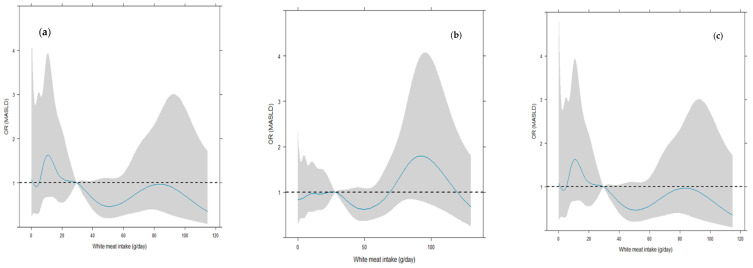
Overall and sex-stratified dose–response curves white meat intake-MASLD. (**a**) Dose–response white meat intake-MASLD, adjusted logistic regression; (**b**) Dose–response white meat intake-MASLD, male, adjusted logistic regression; (**c**) Dose–response white meat intake-MASLD, females, adjusted logistic regression. In cyan is shown the dose–response effect, whereas the 95% confidence bounds are presented in shadowed gray. ORs were significant according to whether their 95% confidence bounds did not involve the value “1”. OR: odds ratio. MASLD: Non-alcoholic fatty liver disease. The models are adjusted for the potential confounders included in the DAG-related minimal sufficient adjustment set.

Figure 1 shows as the overall dose–response effect of “white meat intake—MASLD” relationship is swinging around OR = 1, that is, absence of effect.

The shaded area represents the confidence bands (i.e., confidence intervals for each OR value). For the statistical significance, we judged the OR as significant according to whether its 95% CI involved the value “1”. It is worth pointing out that a large 95% CI indicated a small sample size for corresponding values of white meat intake. As for the sex-stratified trends, an approximately decreasing trend was discovered in males, with small-scale fluctuations, whereas an increasing trend was observed in females. In confirmation of this, the first plot in Figure S1 shows that the sex effect (OR, 1 = male) decreases in relation to the intake, and is significantly bigger than 1 between 5 and 30 g/die.

Regarding the interaction with age and BMI in the overall sample, no significant effects were revealed although the spline function had variability. However, after sex stratification, the estimated dose–response relationships provided a more regular shape, as shown in Figure S2.

Finally, on in-depth analysis linking white meat consumption with “grains” foods, Table S3 shows the statistics for each food across quartile-related white meat intake strata. Notably, when white meat intake increased by quartiles, mean values of “grains” increased and the MASLD proportion decreased. In addition, Figure S3 shows the estimated dose–response relationships, white meat intake-MASLD, both overall and stratified by sex, taking into consideration the “grains” group as moderator: notably, within males a slight protective effect (OR~0.975) from white meat consumption was shown between 100 and 275 g/die of “grains” intake.

Notably, regarding the number of knots of the cubic spline function, when the fitting did not converge, we reduced the number by removing the extreme percentile knots in which the sample size was less. Accounting for this, the interaction dose–response models took fewer knots into consideration.

As for confounders, the DAG-related minimal sufficient adjustment set for the direct effect of white meat intake on MASLD encompassed sex, age, education, BMI, smoking habit, diabetes, total cholesterol, processed meat, red meat, alcohol, soft drinks, daily Kcal intake, fruits, vegetables, legumes, grains, dairy products, sugar foods, fried foods, fish, eggs and margarine (the DAG code using R/dagitty is included in the Appendix in the Supplementary Materials). Only the daily Kcal intake covariate was removed from the set, because it returned a VIF > 5 in the statistical analysis step.

## 4. Discussion

The present study, performed in a population of 1192 subjects belonging to the municipality of Putignano (NUTRIHEP sample), with an average age of 55 years and predominantly overweight, was first addressed to examine the possibility of a dose–response relationship between white meat consumption and the prevalence of MASLD.

A dose–response relationship was not revealed in the whole population, but an important effect of sex was evident. In particular, by increasing the intake, an unfavorable influence of white meat on MASLD was significantly revealed in females, whereas a protective effect of white meat was detectable in males. This is the first report concerning the possible influence of sex on the relationship between white meat consumption and MASLD, but we do not have a clear explanation for these results; however, it is known that prolactin and sex hormones may change the interaction between food and hepatocyte metabolism [36]. Concerning the alternative potential mechanisms to explain the sex differences in the relationship between white meat intake and MASLD, it may well be that nutrients present in white meat may change SHBG levels and consequently free levels of sex hormones differently in men and women; it is also possible that nutrients present in white meat influence the activity of sex chromosomes in hepatocytes, influencing their metabolism. Moreover, we cannot exclude that white meat may derive from animals treated with hormones. These are all simple hypotheses, but further studies are needed to answer to all of these questions. All of this information might be useful for future dietary recommendations.

Sex was shown to exert other effects in this study, such as influencing the preference for specific foods. In fact, men ate a higher amount of poultry, chicken skin and rabbit, and a lower amount of meatballs and white meat hamburger. These results are in line with studies showing the importance of sex when considering the dietary intake assessment in women and in men [37].

Interestingly, differences of food intake associated with sex may reflect differences of the function of specific brain areas [38].

Since men consumed more poultry and simultaneously were less prone to MASLD, it should be noted that poultry meat is a food with higher nutritional quality, highly digestible noble proteins, polyunsaturated fats, B vitamins as well as minerals such as iron, zinc and copper, suggesting that men are indirectly more protected than women from malnutrition.

When consumed in adequate quantities as part of a balanced diet, the fat in white meat (1% in the leanest cuts, such as chicken breast, up to a maximum of 17% in chicken wings and thighs) also plays a number of important roles: it provides ‘essential fatty acids’, such as linoleic and alpha-linolenic acids, fat-soluble vitamins A, D, E, K, promotes satiety and lowers the overall glycaemic load of meals. Accordingly, several studies support the association between poultry consumption and a reduced risk of developing cardiovascular disease, overweight and obesity, insulin resistance and type 2 diabetes mellitus [39].

Age has been shown to change the significance of the association between white meat and MASLD, and increasing age was associated with lower intake of white meat. This result is in line with a higher prevalence of sarcopenia in older people [40].

At variance with sex and age, BMI did not change the relationship between white meat and MASLD, suggesting that body fat distribution, more than body fatness per se, may change this association. Unfortunately, we did not measure body fat distribution in the people enrolled into the study.

As a further interesting result, educational level was paralleled by the intake of white meat: this evidence might suggest that educational level influences health status. This suggestion is reinforced by the finding that higher consumption of white meat was also associated with intake of vegetables, with a magnitude bigger than the association with red meat intake, for example. Lastly, the intake of white meat was paralleled by the consumption of all other sources of proteins such as eggs, fish, other kinds of meat (red and processed) and legumes, suggesting that variations in the intake of white meat would correspond to variations in protein consumption. Concerning the finding that higher intake of white meat was associated with higher consumption of proteins in this study, it should be noted that an inverse association between liver fat content and daily protein intake was shown in adults with metabolic syndrome [41], and this correlation persisted after adjustment with percent of body fat in these subjects. As a matter of fact, recent studies have shown that the development of MASLD is associated with lipid accumulation, oxidative stress, endoplasmic reticulum stress, and lipotoxicity [42].

### Strength and Limitation

The strengths of the present study include a well-defined Italian Mediterranean population and a study sample based on survey data. Thus, we collected information on dietary, socioeconomic, and lifestyle factors using standardized and validated questionnaires, allowing us to minimize sources of bias and confounding. In addition, in this specific geographic location, the population is very “conservative” about eating habits, with consistent behaviour across age groups.

Concerning the limitations, the absence of a validation dataset and the measurement error were not taken into account. Recall may be significantly affected by potential recall bias, introduced by dependence on self-reported dietary data. Thus, it is worth pointing out that the generalisability of the results may be affected by the fact that it does not fully represent the diversity of global populations. Moreover, extreme values of white meat intake may not be representative because of the small sample size. Finally, the observed associations cannot be completely excluded from the potential influence of unmeasured confounding factors. In the end, since the study is observational, not interventional, we prefer not to provide suggestions on the liver-protective diet, but rather to shed light in an explorative way on the association between white meat intake and MASLD.

## 5. Conclusions

Our data suggest that white meat does not have a clear-cut independent effect on MASLD, but sex may influence the development of MASLD, with an increasing unfavorable effect of white meat in women, and a favorable effect of this food in men. Moreover, sex seems to influence the consumption of different types of white meat. Further research is needed to clarify the mechanism between white meat intake and the risk of MASLD, as well as for having a healthy, balanced diet that includes a variety of targeted lean proteins.

## Figures and Tables

**Table 1:** Preliminary statistics.

Variables	Overall (n = 1192)	Males (n = 509, 42.7%)	Females (n = 683, 57.3%)	* *p*-Value Males vs. Females
	Mean ± SD or n (%)	Mean ± SD or n (%)	Mean ± SD or n (%)	
***Exposure***				
*White meat intake (g/die)*	*34.07 ± 26.33*	*35.81 ± 28.68*	*32.77 ± 24.37*	*0.053*
White stewed meat (g/die)	0.925 ± 2.445	1.068 ± 2.841	0.819 ± 2.099	*0.095*
Roast white meat (g/die)	2.407 ± 5.534	2.399 ± 4.68	2.412 ± 6.096	0.968
White cutlet (g/die)	0.808 ± 1.803	0.804 ± 1.793	0.811 ± 1.811	0.946
White meat slices (g/die)	1.42 ± 4.176	1.255 ± 3.022	1.542 ± 4.859	0.209
Rare white meat steak (g/die)	0.362 ± 2.18	0.654 ± 2.958	0.144 ± 1.293	**<0.001**
Medium white meat steak (g/die)	1.363 ± 3.48	1.663 ± 3.945	1.139 ± 3.072	**0.013**
Well done white meat steak (g/die)	0.847 ± 2.534	0.565 ± 1.935	1.057 ± 2.884	**<0.001**
White meat hamburger (g/die)	0.744 ± 1.772	0.562 ± 1.399	0.879 ± 1.996	**0.001**
White meat meatballs (g/die)	1.331 ± 2.513	1.066 ± 2.161	1.528 ± 2.732	**0.001**
Chicken thigh (g/die)	5.983 ± 13.98	6.551 ± 15.929	5.559 ± 12.341	0.243
Chicken breast (g/die)	4.748 ± 11.464	4.508 ± 11.596	4.926 ± 11.371	0.535
Chicken—other parts (g/die)	2.175 ± 7.367	1.621 ± 6.747	2.587 ± 7.776	**0.022**
Poultry (g/die)	7.612 ± 14.797	9.294 ± 16.958	6.358 ± 12.824	**0.001**
Chicken Skin (g/die)	0.418 ± 0.681	0.5684 ± 0.776	0.305 ± 0.576	**<0.001**
Rabbit (g/die)	2.925 ± 4.456	3.23 ± 4.713	2.698 ± 4.243	**0.044**
***Outcome***				
MASLD (yes/no)	587/605 (49.2/50.7)	278/231 (54.6/45.4)	309/374 (45.2/54.8)	**0.001**
***Confounders***				
Age (y)	54.65 ± 14.37	55.1 ± 15.19	54.32 ± 13.738	0.361
Education (high School or more: yes/no)	570/622 (47.8/52.2)	254/255 (49.9/50.1)	316/367 (46.3/53.7)	0.236
Body Mass Index (kg/m^2^)	27.61 ± 4.981	27.93 ± 4.413	27.37 ± 5.357	*0.050*
Smoke (yes/no)	146/1046 (12.2/87.8)	81/428 (15.9/84.1)	65/618 (9.5/90.5)	**<0.001**
Diabetes (yes/no)	80/1112 (6.7/93.3)	37/472 (7.3/92.7)	43/640 (6.3/93.7)	0.506
Cholesterol (mg/dL)	191.4 ± 36.061	187.5 ± 37.095	194.4 ± 35.012	**0.001**
Daily caloric intake (kcal/die)	2045 ± 737.13	2207 ± 780.52	1924.2 ± 678.87	**<0.001**
Alcohol (g/die)	10.41 ± 19.96	18.499 ± 27.28	4.38 ± 7.522	**<0.001**
Dairy foods (g/die)	118.33 ± 105.51	112.2 ± 101.16	122.9 ± 108.48	*0.078*
Fish (g/die)	37.91 ± 25.59	38.37 ± 26.74	37.57 ± 24.71	0.598
Fruits (g/die)	297 ± 169.19	311 ± 187.6	286.5 ± 153.4	**0.016**
Fried foods (g/die)	6.45 ± 8.76	7.38 ± 8.71	5.758 ± 8.73	**0.001**
Eggs (g/die)	19.77 ± 15.34	19.37 ± 18.37	20.06 ± 12.62	0.468
Grains (g/die)	183.6 ± 97.98	203.7 ± 108.02	168.7 ± 86.89	**<0.001**
Legumes (g/die)	38.58 ± 31.36	42.57 ± 36.15	35.6 ± 26.9	<0.001
Margarine (g/die)	0.048 ± 0.339	0.05 ± 0.352	0.046 ± 0.330	0.871
Soft drink (g/die)	67.8 ± 144.36	70.23 ± 160.15	65.98 ± 131.46	0.625
Sugar foods (g/die)	82.23 ± 67.13	84.36 ± 71.85	80.64 ± 63.38	0.353
Vegetables (g/die)	205.5 ± 111.47	193.5 ± 106.21	214.4 ± 14.48	**0.001**
*Red meat intake (g/die)*	*44.86 ± 31.23*	*51.39 ± 34.22*	*39.99 ± 27.85*	**<0.001**
Meat sauce on pasta (g/die)	2.531 ± 5.119	3.616 ± 6.536	1.722 ± 3.522	**<0.001**
Meat sauce on rice (g/die)	1.617 ± 2.638	1.709 ± 3.161	1.549 ± 2.168	0.327
Meat broth (g/die)	2.699 ± 7.135	3.114 ± 8.063	2.39 ± 6.345	*0.094*
Red stewed meat (g/die)	1.913 ± 4.197	2.341 ± 4.799	1.593 ± 3.656	**0.003**
Roast red meat (g/die)	4.556 ± 7.041	5.523 ± 8.248	3.836 ± 5.889	<0.001
Red meat boil (g/die)	2.045 ± 4.885	2.348 ± 5.796	1.819 ± 4.067	*0.078*
Red cutlet (g/die)	1.687 ± 3.031	1.936 ± 3.634	1.501 ± 2.475	**0.020**
Red meat slices (g/die)	2.606 ± 5.463	3.176 ± 6.896	2.182 ± 4.034	**0.003**
Rare red meat steak (g/die)	0.751 ± 3.667	1.238 ± 4.008	0.389 ± 3.347	**<0.001**
Medium red meat steak (g/die)	2.695 ± 5.351	3.428 ± 6.18	2.149 ± 4.568	**<0.001**
Well done red meat steak (g/die)	1.661 ± 4.468	1.3 ± 4.018	1.93 ± 4.761	**0.013**
Red meat hamburger (g/die)	1.494 ± 2.898	1.319 ± 2.58	1.625 ± 3.11	*0.064*
Red meat meatballs (g/die)	3.004 ± 4.510	2.566 ± 3.939	3.331 ± 4.87	**0.003**
Animal fat (g/die)	0.568 ± 0.894	0.754 ± 1.075	0.429 ± 0.699	**<0.001**
Fatty meat (g/die)	6.762 ± 7.811	7.521 ± 7.803	6.196 ± 7.775	**0.003**
Sheep meat (g/die)	2.263 ± 3.986	2.805 ± 4.415	1.859 ± 3.585	**<0.001**
Horse meat (g/die)	4.312 ± 8.53	4.635 ± 8.968	4.072 ± 8.186	0.266
Liver (g/die)	1.239 ± 3.157	1.504 ± 3.567	1.042 ± 2.799	**0.015**
Giblets (g/die)	0.456 ± 1.22	0.562 ± 1.276	0.377 ± 1.172	**0.011**
*Processed meat intake (g/die)*	*28.4 ± 27.87*	*33.16 ± 29.92*	*24.86 ± 25.7*	**<0.001**
Cotechino o zampone (g/die)	8.37 ± 8.863	9.186 ± 9.689	7.761 ± 8.148	**0.007**
Canned meat (g/die)	0.494 ± 2.069	0.597 ± 2.285	0.418 ± 1.889	0.152
Cured meat sandwich (g/die)	6.953 ± 13.581	9.288 ± 15.691	5.213 ± 11.471	**<0.001**
Ham (g/die)	2.825 ± 4.339	2.735 ± 4.025	2.892 ± 4.561	0.530
Lean ham (g/die)	3.385 ± 5.376	4.065 ± 6.499	2.878 ± 4.29	**<0.001**
Cured meat, sausages (g/die)	1.438 ± 3.148	1.602 ± 2.638	1.317 ± 3.47	0.107
Mortadella (g/die)	1.289 ± 2.857	1.597 ± 3.021	1.06 ± 2.708	**0.001**
Bresaola (g/die)	1.854 ± 3.405	1.823 ± 3.535	1.876 ± 3.307	0.792
Soppressata (g/die)	0.932 ± 2.185	1.251 ± 2.641	0.695 ± 1.735	**<0.001**
Other cured meat (g/die)	0.639 ± 2.424	0.734 ± 2.993	0.568 ± 1.890	0.273
Fatty ham (g/die)	0.221 ± 0.369	0.277 ± 0.397	0.178 ± 0.34	**<0.001**

Note. SD: standard deviation, n: sample size, y: years, MASLD: Metabolic dysfunction-associated steatotic liver disease; In **bold** the significant results (*p* < 0.05), in *italic* the suggestive results (0.05 < *p* < 0.10). * Two-tail unpaired *T*-test or Chi-square test, as appropriate.

**Table 2:** Statistics for quartiles of white meat intake, overall and by sex.

	Overall					Males					Females					Males vs. Females
	Min	1st q	2nd q	3rd q	Max	Min	1st q	2nd q	3rd q	Max	Min	1st q	2nd q	3rd q	Max	Mann–Whitney *p*-Value
**White meat (g/die)**	**0**	**16.7**	**28.2**	**44.6**	**245**	**0**	**17.4**	**29.7**	**47.6**	**245**	**0**	**16.4**	**27**	**43.2**	**197.6**	0.108
White stewed meat (g/die)	0	0.0	0.0	0.9	28.7	0	0.0	0.0	0.9	28.7	0	0.0	0.0	0.8	26.4	0.646
Roast white meat (g/die)	0	0.0	0.6	2.5	85.7	0	0.0	0.6	2.7	42.9	0	0.00	0.70	2.25	85.7	0.818
White cutlet (g/die)	0	0.0	0.0	0.9	17.6	0	0.0	0.0	0.9	16.1	0	0.0	0.0	0.9	17.6	0.397
White meat slices (g/die)	0	0.0	0.0	1.1	56.3	0	0.0	0.0	1.1	27.4	0	0.0	0.0	1.2	56.3	0.318
Rare white meat steak (g/die)	0	0.0	0.0	0.0	45.7	0	0.0	0.0	0.0	45.7	0	0.0	0.0	0.0	25.3	**<0.001**
Medium white meat steak (g/die)	0	0	0	1	40	0	0.0	0.0	1.2	23.4	0	0.0	0.0	0.7	40	0.127
Well done white meat steak (g/die)	0	0.0	0.0	0.0	26.9	0	0.0	0.0	0.0	16.1	0	0.00	0.00	0.65	26.9	**<0.001**
White meat hamburger (g/die)	0	0.0	0.0	0.7	17	0	0.0	0.0	0.6	13.4	0	0.0	0.0	0.9	17.1	**0.002**
White meat meatballs (g/die)	0	0.0	0.3	1.5	25	0	0.0	0.0	1.3	22.4	0	0.0	0.5	1.9	25	**<0.001**
Chicken thigh (g/die)	0	0.0	0.0	6.9	165.3	0	0.0	0.0	6.9	165.3	0	0.0	0.0	6.9	102.3	0.770
Chicken breast (g/die)	0	0.0	0.0	0.0	102.3	0	0.0	0.0	0.0	76.7	0	0.0	0.0	1.3	102.3	0.127
Chicken—other parts (g/die)	0	0.0	0.0	0.0	61.7	0	0.0	0.0	0.0	61.7	0	0.0	0.0	0.0	51.1	**<0.001**
Poultry (g/die)	0	0.0	0.0	11.9	153.4	0	0.0	0.0	14.9	153.4	0	0.0	0.0	9.4	102.3	**0.002**
Chicken Skin (g/die)	0	0.0	0.1	0.6	6.2	0	0.0	0.3	0.8	6.2	0	0.0	0.0	0.4	5.1	**<0.001**
Rabbit (g/die)	0	0.0	1.3	4.0	34.3	0	0.3	1.3	4.0	34.3	0	0.0	1.0	4.0	34.3	**0.002**

Note. In **bold** the significant results (*p* < 0.05). Min: minimum. Max: maximum. q: quartile.

**Table 3:** Results of logistic regression models on MASLD: raw, adjusted and quartiles categorization effects.

	Overall (n = 1197, 587 MASLD)			Males (n = 509, 278 MASLD)			Females (n = 653, 309 MASLD)		
	OR *p*-Value 95% CI	OR *p*-Value 95% CI	OR * p-Value 95% CI	OR *p*-Value 95% CI	OR *p*-Value 95% CI	OR * p-Value 95% CI	OR *p*-Value 95% CI	OR *p*-Value 95% CI	OR * p-Value 95% CI
	Raw Models	Adjusted Models	Quartiles Categorized Exposure *	Raw Models	Adjusted Models	Quartiles Categorized Exposure *	Raw Models	Adjusted Models	Quartiles Categorized Exposure *
White meat (g/die)	*0.996* *0.096* *0.992; 1.001*	0.998 0.545 0.992; 1.004	OR_25–50_ = 1.009 0.964 0.668; 1.524 OR_50–75_ = 0.914 0.679 0.599; 1.395 OR_75–100_ = 0.914 0.699 0.579; 1.442	**0.987** **<0.001** **0.981; 0.994**	**0.985** **0.005** **0.976; 0.995**	OR_25–50_ = 0.662 0.221 0.342; 1.281 **OR_50–75_ = 0.502** **0.041** **0.259; 0.971** **OR_75–100_ = 0.319** **0.002** **0.153; 0.665**	1.003 0.276 0.997; 1.009	*1.008* *0.072* *0.999; 1.017*	OR_25–50_ = 1.338 0.305 0.766; 2.334 OR_50–75_ = 1.163 0.609 0.652; 2.073 *OR_75–100_ = 1.780* *0.067* *0.960; 3.298*
White stewed meat (g/die)	1.001 0.979 0.955; 1.048	0.983 0.592 0.926; 1.044	^ OR_75–100_ = 0.879 0.466 0.621; 1.244	*0.943* *0.088* *0.883; 1.008*	0.940 0.142 0.865; 1.02113	^ OR_75–100_ = 0.923 0.765 0.547; 1.557	*1.073* *0.072* *0.993; 1.159*	1.051 0.371 0.943; 1.171	^ OR_75–100_ = 1.016 0.946 0.637; 1.622
Roast white meat (g/die)	0.998 0.875 0.978; 1.019	*1.027* *0.065* *0.999; 1.057*	^ OR_50–100_ = 0.977 0.878 0.731; 1.306	0.970 0.126 0.934; 1.008	1.014 0.588 0.964; 1.066	^ OR_50–100_ = 1.094 0.694 0.698; 1.714	1.011 0.405 0.985; 1.036	*1.036* *0.067* *0.997; 1.075*	^ OR_50–100_ = 0.918 0.668 0.619; 1.360
White cutlet (g/die)	0.982 0.576 0.922; 1.046	0.994 0.884 0.916; 1.077	^ OR_75–100_ = 0.856 0.380 0.607; 1.209	*0.908* *0.066* *0.819; 1.006*	0.930 0.297 0.812; 1.066	^ OR_75–100_ = 0.666 0.141 0.387; 1.144	1.037 0.382 0.954; 1.128	1.048 0.417 0.936; 1.172	^ OR_75–100_ = 1.047 0.841 0.664; 1.652
White meat slices (g/die)	**0.953** **0.009** **0.919; 0.988**	0.974 0.166 0.940; 1.010	**^ OR_75–100_ = 0.664** **0.022** **0.467; 0.943**	**0.895** **0.002** **0.833; 0.963**	**0.896** **0.012** **0.823; 0.976**	^ OR_75–100_ = 0.695 0.184 0.407; 1.188	0.976 0.204 0.942; 1.012	0.991 0.666 0.956; 1.029	*^ OR_75–100_ = 0.621* *0.055* *0.380; 1.013*
Rare white meat steak (g/die)	0.982 0.532 0.931; 1.037	0.982 0.668 0.907; 1.064	^ OR_95–100_ = 1.014 0.965 0.516; 1.992	0.955 0.196 0.892; 1.023	0.972 0.581 0.877; 1.076	^ OR_95–100_ = 1.114 0.842 0.383; 3.242	1.027 0.649 0.913; 1.156	0.998 0.976 0.875; 1.138	^ OR_96–100_ = 0.931 0.887 0.347; 2.501
Medium white meat steak (g/die)	0.993 0.696 0.961; 1.026	0.997 0.915 0.958; 1.038	^ OR_75–100_ = 0.871 0.418 0.625; 1.215	0.970 0.192 0.928; 1.015	0.978 0.437 0.925; 1.034	^ OR_75–100_ = 0.658 0.115 0.391; 1.107	1.012 0.610 0.964; 1.063	1.022 0.514 0.956; 1.094	^ OR_75–100_ = 1.138 0.568 0.729; 1.775
Well done white meat steak (g/die)	0.977 0.328 0.934; 1.023	0.965 0.262 0.909; 1.026	^ OR_90–100_ = 0.828 0.443 0.512; 1.340	0.934 0.153 0.851; 1.025	0.916 0.152 0.812; 1.033	^ OR_90–100_ = 0.628 0.213 0.302; 1.305	1.001 0.954 0.950; 1.055	0.983 0.650 0.915; 1.056	^ OR_90–100_ = 1.089 0.792 0.575; 2.065
White meat hamburger (g/die)	**0.906** **0.006** **0.844; 0.972**	0.954 0.291 0.876; 1.040	^ OR_75–100_ = 0.759 0.115 0.539; 1.069	**0.833** **0.014** **0.719; 0.964**	*0.862* *0.076* *0.731; 1.016*	^ OR_75–100_ = 0.714 0.214 0.419; 1.214	0.945 0.167 0.872; 1.023	0.995 0.932 0.901; 1.099	^ OR_75–100_ = 0.812 0.377 0.511; 1.289
White meat meatballs (g/die)	0.982 0.453 0.939; 1.028	0.982 0.563 0.924; 1.043	^ OR_75–100_ = 0.822 0.256 0.587; 1.152	0.948 0.214 0.873; 1.030	0.962 0.491 0.863; 1.072	^ OR_75–100_ = 1.048 0.858 0.662; 1.768	1.008 0.765 0.954; 1.065	0.997 0.956 0.925; 1.075	^ OR_75–100_ = 0.935 0.772 0.595; 1.470
Chicken thigh (g/die)	*0.991* *0.061* *0.983; 1.000*	0.995 0.369 0.984; 1.005	^ OR_75–100_ = 0.865 0.409 0.614; 1.219	*0.988* *0.063* *0.977; 1.001*	*0.986* *0.061* *0.972; 1.001*	^ OR_75–100_ = 0.754 0.299 0.442; 1.284	0.994 0.381 0.982; 1.006	1.004 0.607 0.987; 1.021	^ OR_75–100_ = 0.910 0.689 0.572; 1.445
Chicken breast (g/die)	**0.984** **0.004** **0.974; 0.995**	0.996 0.548 0.983; 1.009	^ OR_80–100_ = 0.853 0.404 0.588; 1.238	**0.978** **0.009** **0.963; 0.994**	0.990 0.305 0.972; 1.009	^ OR_80–100_ = 0.869 0.619 0.499; 1.512	0.989 0.143 0.976; 1.003	1.003 0.755 0.984; 1.021	^ OR_80–100_ = 0.932 0.789 0.555; 1.563
Chicken—other parts (g/die)	1.013 0.104 0.997; 1.029	1.010 0.317 0.990; 1.030	^ OR_95–100_ = 1.649 0.173 0.802; 3.387	1.006 0.652 0.979; 1.032	1.009 0.535 0.979; 1.042	^ OR_95–100_ = 1.995 0.167 0.748; 5.313	*1.010* *0.051* *0.999; 1.040*	1.011 0.435 0.984; 1.038	^ OR_95–100_ = 1.655 0.294 0.646; 4.245
Poultry (g/die)	1.005 0.176 0.997; 1.013	1.001 0.921 0.990; 1.011	^ OR_75–100_ = 1.044 0.803 0.743; 1.467	0.998 0.794 0.988; 1.008	0.997 0.716 0.982; 1.012	^ OR_75–100_ = 0.845 0.563 0.478; 1.494	*1.011* *0.060* *0.999; 1.023*	1.006 0.445 0.991; 1.022	^ OR_75–100_ = 1.074 0.753 0.649; 1.687
Chicken Skin (g/die)	0.964 0.668 0.816; 1.139	0.896 0.386 0.701; 1.148	^ OR_75–100_ = 0.851 0.402 0.584; 1.240	*0.802* *0.060* *0.637; 1.009*	0.793 0.180 0.566; 1.112	*^ OR_75–100_ = 0.591* *0.061* *0.340; 1.023*	1.081 0.558 0.832; 1.403	1.039 0.849 0.694; 1.558	^ OR_75–100_ = 1.044 0.870 0.623; 1.749
Rabbit (g/die)	**1.029** **0.031** **1.002; 1.056**	1.007 0.638 0.975; 1.041	^ OR_50–100_ = 1.135 0.401 0.844; 1.525	1.010 0.580 0.973; 1.049	0.989 0.681 0.942; 1.040	^ OR_50–100_ = 1.091 0.710 0.690; 1.726	**1.042** **0.028** **1.004; 1.081**	1.020 0.387 0.975; 1.068	^ OR_50–100_ = 1.119 0.578 0.753; 1.662

Note. In **bold** the significant results (*p* < 0.05), in *italic* the suggestive ones (0.05 < *p* < 0.10). 95% CI: 95% confidence interval.* The models are adjusted for the potential confounders included in the DAG-related minimal sufficient adjustment set. ^ Zero-inflated predictor: categorized only on median or 3rd quartile, by using the lower category as reference. If this categorization returned non-different ‘breaks’, percentiles were applied (e.g., 95%).

## Data Availability

The original contributions presented in this study are included in the article. Further inquiries can be directed to the corresponding author.

## Supplementary Materials

The following supporting information can be downloaded at: https://www.mdpi.com/article/10.3390/nu16183094/s1, Figure S1: Interaction effects of sex, age and BMI with white meat intake on MASLD; Figure S2: Interaction effects of age and BMI with white meat intake on MASLD by sex; Figure S3: Interaction effects of grains with white meat intake on MASLD, overall and by sex; Table S1: Food groups confounders; Table S2: Association between exposure and confounders. Table S3: Subject grain (carbohydrates) profiling by daily white meat intake quartiles stratification; Appendix: R/dagitty code on the Direct Acyclic Graph (DAG) for theory driven minimal sufficient adjustment set of the confounders of the direct effect of the white meat intake on MASLD.
