# Supplementary material for: A Dose–Response Study on the Relationship between White Meat Intake and Metabolic Dysfunction-Associated Steatotic Liver Disease (MASLD) in Southern Italy: Results from the Nutrihep Study
Source: Nutrients. 2024 Sep 13;16(18):3094. doi: 10.3390/nu16183094 (PMC11435003; doi:10.3390/nu16183094)
Supplement: Supplementary file 1 [file nutrients-16-03094-s001.zip › nutrients-3173461-supplementary.pdf]

Supplementary

Table S1 – Food groups confounders

Grains	Vegetables	Legumes	Dairy products	Fried foods	Fish	Eggs	Fruits	Soft drinks	Sugar foods	Margarine
PASTASCIUTTA	TOMATO PASTA	PASTA LEG-UMES	WHITE BUTTER	FISH BASTONES	SHRIMP	EGGS	APPLE	ORANGE SQUASH	HONEY	WHITE MAR-GARINE
EGG PASTA	VEGETABLES PASTA	LEGUMES RICE	TOMATO BUTTER	FRYED POTA-TOES	OCTOPES-SEPS	MAYO	PEAR	FRUIT JUICES	SUGAR MILK	MARGARINE TOMATO
FILLED PASTA	VEGETABLES RICE	MINESTRA OF LEGUMES	BUTTER RAGU	POTATO CROQUETTES	MUSSELS-CLAMS	OME-LETTE	BANANA	SOFT DRINKS	SWEETENED COFFEE	MARGARINA RAGU
RICE	MINESTRON	DRIED BEANS	BUTTER VEGETA-BLES		PRESERVED FISH		KIWI		SUGAR YO-GURT	VEGETABLE MARGARINE LEGUMS
RICE SALAD	VEGETABLE BROTH	FRESH BEANS FRIED	BUTTER MINE-STRON		BOXED FISH		ORANGES GRAPEFRUIT		SUGAR CAFE-LATTE	MARGARINE MINESTRON
PASTA IN SOUP	TOMATOES IN CANNED	CANNED BEANS	ADDED BUTTER		SOGLIOLA FISH		MANDARINS		SUGAR TEA/INFU-SIONS	WET MARGA-RINE
POLENTA	TOMATOES END STAGE	DRIED FA-VES	GRATED CHEESE		SARDINES		GRAPES		SQUEEZED SUGAR	FRIED MAR-GARINE
PIZZA CUT	SALAD	FRIED FRESH FAVORS	FONTINA FONTAL		TROUT		PEACHES		BISCUITS	
PIZZA from PIZ-ZERIA	RAW PEPPER	TINNED BROAD BEANS	PECORINO CACIOTTA		SALMON		APRICOTS		BRIOCHES	
HOMEMADE PIZZA	RAW ONIONS	DRIED LEN-TILS	EMMENTHAL GROVIERA		SPADISH		PLUMS		MARMALADE	

CHIPS OR SAL-ADS	CRUCIBLE CAR-ROFS	FRESH FRIED LENTILS	CACIOCAVAL		OTHER TYPES OF FISH		STRAWBER-RIES		NUTELLA	
COMMON BREADS	CRUCIBLE FEN-NEL	LENTILS IN SQUASH	PARMIGIANO				MELON		FILLED CAKE	
WHITE BREAD	CRUDIED SE-DANS	DRIED CHICKPEAS	OTHER TYPES OF MATURED CHEESE				FRUIT SALAD		UNSTUFFED CAKE	
WHOLE-GRAIN SANDWICHES	SOYBEANS	FROZEN CHICKPEAS	SOTTILETS						SPOON PIE	
WHOLE GRAIN CRACKERS	CRUCIBLE CAB-BAGE	CANNED CHICKPEAS	PHILADELFIA						DRY PASTRIES	
WHOLE BREAD	CRUDE CAR-ROTS	DRIED PEAS	CRESCENCE						FILLED COOKIES	
CONDITIONED BREADS	ONION OR LEEK SAUCES	FRESH FRO-ZEN PEAS	MOZZARELLA						CHOCOLATE	
TOASTS	COOKED CAR-ROTS	CANNED PEAS	TALEGGIO						CARAMELS	
CORN FLAKES	BROCCOLI		GORGONZOLA						SUMMER ICE CREAM	
MUESLI OAT-MEAL FRITTERS	BRUSSELS SPROUTS		ROBIOLA						ICE CREAM IN WINTER	
WHOLE GRAINS	CAULIFLOWER		RICOTTA							
SEEDS	RAPA SEEDS		OTHER TYPES OF SPRUCE CHEESES							
	BLACK CAB-BAGE		WHITE WHOLE MILK							
	VIRLS		PARTIALLY SKIMMED WHITE MILK							

	SPINACH		SKIMMED WHITE MILK							
	COOKED PEP-PER		WHOLE FRUIT YOGURT							
	AUBERGINE		FRUIT YOGURT SKIMMED							
	GREEN BEAN		YOGHURT OTHER WHOLE							
	ZUCCHINI		YOGURT OTHER LOW-FAT							
	COOKED ARTI-CHOKES		BUTTER							
	COOKED FEN-NEL		FRYED BUTTER							
	RED BEETS		MOIST BUTTER							
	COOKED ON-IONS		SANDWICHES WITH CHEESE							
	VEGETABLE PIES									
	SANDWICHES WITH VEGETA-BLES									

Table S2. Association between exposure and confounders

1st part	Red meat	Processed meat	AGE	Sex (1=male)	Education (1= High School or more)	BMI	Smoke (1=yes)	Diabetes	Cholesterol	Alcohol	SOFT DRINK
**White meat**	**r=0.348**<br>**P<0.001**<br>**95%CI=[0.297; 0.397]**	**r =0.276**<br>**<0.001**<br>**0.222; 0.327**	**r =-0.278**<br>**P <0.001**<br>**-0.330; -0.225**	*MD=3.045*<br>*P=0.053*<br>*-0.049; 6.139*	**MD=3.621**<br>**P=0.017**<br>**0.638; 6.605**	r =0.045<br>0.118<br>-0.011; 0.101	*MD=5.060*<br>*P=0.061*<br>*-0.234; 10.355*	**MD=-4.739**<br>**<0.001**<br>**-35.496; -32.503**	r =0.015<br>0.588<br>-0.041; 0.072	r =0.047<br>0.102<br>-0.009; 0.103	**r =0.096**<br>**<0.001**<br>**0.040; 0.152**
**Red meat**	-	**r =0.324**<br>**<0.001**<br>**0.272; 0.37**	**r =-0.128**<br>**<0.001**<br>**-0.184; -0.072**	**MD=11.401**<br>**<0.001**<br>**7.763; 15.038**	MD=-2.002<br>0.268<br>-5.553; 1.549	r =0.030<br>0.286<br>0.087; -0.025	**MD=9.985**<br>**0.002**<br>**3.650; 16.321**	r =-4.066<br>P=0.267<br>-11.303; 3.169	r =-0.017<br>0.551<br>-0.073; 0.039	**r =0.195**<br>**<0.001**<br>**0.139; 0.249**	**r =0.084**<br>**0.004**<br>**0.027; 0.140**
**Processed meat**	-	-	**r =-0.329**<br>**<0.001**<br>**-0.378; -0.277**	**MD=8.298**<br>**<0.001**<br>**5.058; 11.538**	**MD=8.761**<br>**<0.001**<br>**5.613; 11.909**	r =-0.019<br>0.519<br>-0.075; 0.038	**MD=10.212**<br>**<0.001**<br>**4.407; 16.016**	**MD=-8.765**<br>**0.002**<br>**14.202; 3.327**	r=0.016<br>0.559<br>-0.039; 0.073	**r=0.144**<br>**<0.001**<br>**0.088; 0.199**	**r=0.158**<br>**<0.001**<br>**0.102; 0.213**
**AGE**	-	-	-	MD=0.779<br>0.362<br>-0.896; 2.456	**MD=-13.8**<br>**<0.001**<br>**-15.241; -12.359**	**r=0.294**<br>**<0.001**<br>**0.241; 0.345**	**MD=-4.691**<br>**<0.001**<br>**-7.209; -2.171**	**MD=12.645**<br>**<0.001**<br>**10.354; 14.936**	**r=0.064**<br>**0.025**<br>**0.008; 0.121**	**r=0.084**<br>**0.003**<br>**0.028; 0.141**	**r=-0.141**<br>**<0.001**<br>**-0.196; -0.084**
**Sex**	-	-	-	-	X^2= 1.402<br>0.236	*MD=0.556*<br>*0.051*<br>*0.000; 1.112*	X^2=**10.516**<br>**0.001**	X^2= 0.299<br>0.584	**MD=-6.863**<br>**0.001**<br>**-11.023; -2.700**	**MD=14.118**<br>**<0.001**<br>**11.677; 16.560**	MD=4.251<br>0.652<br>-12.821; 21.325
**Education**	-	-	-	-	-	**-2.852**<br>**<0.001**<br>**-3.392; -2.312**	**X^2= 5.033**<br>**0.024**	**X^2= 18.889**<br>**<0.001**	MD=-2.312<br>0.268<br>-6.407; 1.782	MD=-1.392<br>0.237<br>-3.702; 0.917	MD=7.827<br>0.351<br>-8.624; 24.28
**BMI**	-	-	-	-	-	-	MD=-0.640<br>0.123	**MD=4.531**<br>**<0.001**	**r=0.095**<br>**0.001**	r=0.019<br>0.509	r=0.042<br>0.143

							-1.456;<br>0.175	**3.236; 5.825**	**0.038; 0.151**	-0.037;<br>0.075	-0.014; 0.09
**Smoke**	-	-	-	-	-	-	-	$X^2 < 0.001$<br>1	MD=-2.873<br>0.345<br>-8.871; 3.123	**MD=6.746**<br>**<0.001**<br>**3.355;**<br>**10.137**	MD=23.836<br>0.227<br>-15.049;<br>62.721
**Diabetes**	-	-	-	-	-	-	-	-	**MD=-**<br>**17.441**<br>**<0.001**<br>**-25.504; -**<br>**9.377**	MD=2.073<br>0.305<br>-1.915;<br>6.061	MD=-<br>17.222<br>0.298<br>-49.937;<br>15.491
**Cholesterol**	-	-	-	-	-	-	-	-	-	r=-0.018<br>0.516<br>-0.075;<br>0.038	r=0.022<br>0.444<br>-0.034;<br>0.078
**Alcohol**	-	-	-	-	-	-	-	-	-	-	**r=0.126**<br>**<0.001**<br>**0.070; 0.182**
**Soft drink**	-	-	-	-	-	-	-	-	-	-	-
**Energy intake**	-	-	-	-	-	-	-	-	-	-	-
**Dairy foods**	-	-	-	-	-	-	-	-	-	-	-
**Sugar foods**	-	-	-	-	-	-	-	-	-	-	-
**Fruits**	-	-	-	-	-	-	-	-	-	-	-
**Vegetables**	-	-	-	-	-	-	-	-	-	-	-
**Legumes**	-	-	-	-	-	-	-	-	-	-	-
**Grains**	-	-	-	-	-	-	-	-	-	-	-
**Fried foods**	-	-	-	-	-	-	-	-	-	-	-
**Fish**	-	-	-	-	-	-	-	-	-	-	-
**Eggs**	-	-	-	-	-	-	-	-	-	-	-
2nd part											

	Energy intake	Dairy products	Sugar foods	Fruits	Vegetables	Legumes	Grains	Fried foods	Fish	Eggs	Margarine
**White meat**	**r=0.312**<br>**<0.001**<br>**0.260; 0.363**	r=0.010<br>0.705<br>-0.045; 0.067	**r=0.100**<br>**<0.001**<br>**0.044; 0.156**	r=0.004<br>0.896<br>-0.053; 0.060	**r=0.206**<br>**<0.001**<br>**0.151; 0.260**	**r=0.135**<br>**<0.001**<br>**0.077; 0.189**	**r=0.126**<br>**<0.001**<br>**0.070; 0.182**	**r=0.078**<br>**0.006**<br>**0.022; 0.135**	**r=0.259**<br>**<0.001**<br>**0.206; 0.311**	**r=0.214**<br>**<0.001**<br>**0.159; 0.267**	r=-0.018<br>0.517<br>-0.075; 0.038
**Red meat**	**r=0.393**<br>**<0.001**<br>**0.344; 0.440**	r=0.0346<br>0.231<br>-0.022; 0.091	**r=0.105**<br>**<0.001**<br>**0.048; 0.161**	r=-0.021<br>0.479<br>-0.077; 0.036	**r=0.083**<br>**0.003**<br>**0.027; 0.139**	**r=0.134**<br>**<0.001**<br>**0.078; 0.190**	**r=0.249**<br>**<0.001**<br>**0.195; 0.301**	**r=0.239**<br>**<0.001**<br>**0.185; 0.292**	**r=0.130**<br>**<0.001**<br>**0.074; 0.185**	**r=0.120**<br>**<0.001**<br>**0.064; 0.176**	r=0.047<br>0.106<br>-0.009; 0.103
**Processed meat**	**r=0.464**<br>**<0.001**<br>**0.418; 0.507**	r=0.034<br>0.235<br>-0.022; 0.090	**r=0.213**<br>**<0.001**<br>**0.159; 0.267**	*r=-0.050*<br>*0.078*<br>*-0.107; 0.006*	**r=0.091**<br>**0.002**<br>**0.035; 0.147**	r=0.027<br>0.342<br>-0.029; 0.084	**r=0.289**<br>**<0.001**<br>**0.236; 0.340**	**r=0.256**<br>**<0.001**<br>**0.202; 0.308**	**r=0.098**<br>**<0.001**<br>**0.041; 0.154**	**r=0.121**<br>**<0.001**<br>**0.064; 0.176**	r=0.015<br>0.611<br>-0.042; 0.071
**AGE**	**r=-0.181**<br>**<0.001**<br>**-0.235; -0.125**	**r=0.082**<br>**0.005**<br>**0.025; 0.138**	**r=-0.189**<br>**<0.001**<br>**-0.243; -0.134**	**r=0.201**<br>**<0.001**<br>**0.146; 0.255**	**r=-0.121**<br>**<0.001**<br>**-0.176; -0.064**	r=0.022<br>0.439<br>-0.034; 0.079	**r=-0.113**<br>**<0.001**<br>**-0.169; -0.057**	**r=-0.097**<br>**<0.001**<br>**-0.152; -0.040**	**r=-0.121**<br>**<0.001**<br>**-0.176; -0.064**	**r=-0.096**<br>**<0.001**<br>**-0.152; -0.039**	*r=0.052*<br>*0.072*<br>*-0.004; 0.109*
**Sex**	**MD=283.01**<br>**<0.001**<br>**198.11; 367.905**	*MD=-10.7711*<br>*0.078*<br>*-22.760; 1.218*	MD=3.718<br>0.353<br>-4.137; 11.574	**MD=24.457**<br>**0.016**<br>**4.484; 44.429**	**MD=-20.948**<br>**0.001**<br>**-33.565; 8.331**	**MD=6.969**<br>**<0.001**<br>**3.231; 10.707**	**MD=34.988**<br>**<0.001**<br>**23.548; 46.428**	**MD=1.621**<br>**0.002**<br>**0.6193; 2.624**	MD=0.798<br>0.598<br>-2.177; 3.774	MD=-0.687<br>0.468<br>-2.545; 1.171	MD=0.003<br>0.872<br>-0.036; 0.042
**Education**	**MD=116.989**<br>**0.006**<br>**33.370; 200.607**	MD=-7.595<br>0.213<br>-19.546; 4.356	**MD=12.039**<br>**0.001**<br>**4.437; 19.640**	**MD=-29.457**<br>**0.003**<br>**-48.680; -10.234**	**MD=21.254**<br>**0.001**<br>**8.585; 33.923**	MD=-1.037<br>0.566<br>-4.590; 2.515	**MD=12.512**<br>**0.027**<br>**1.411; 23.614**	**MD=-29.457**<br>**0.003**<br>**-48.680; -10.234**	**MD=4.655**<br>**0.002**<br>**1.751; 7.559**	**MD=1.987**<br>**0.027**<br>**0.223; 3.751**	MD=-0.027<br>0.159<br>-0.065; 0.010
**BMI**	r=-0.032<br>0.266<br>-0.088; 0.024	r=0.022<br>0.433<br>-0.034; 0.079	**r=-0.119**<br>**<0.001**	**r=0.067**<br>**0.019**<br>**0.011; 0.124**	r=0.022<br>0.439<br>-0.034; 0.079	r=0.047<br>0.101	r=-0.021<br>0.466	r=-0.020<br>0.482<br>-0.077; 0.036	r=0.011<br>0.701<br>-0.045; 0.067	r=-0.021<br>0.464	r=0.030<br>0.287

			**-0.175; -<br>0.063**			-0.009;<br>0.104	-0.077;<br>0.035			-0.077;<br>0.035	-0.026;<br>0.087
**Smoke**	**MD=225.018<br>0.002<br>82.176; 367.86**	MD=-5.353<br>0.567<br>- 23.791;<br>13.085	MD=5.058<br>0.467<br>-8.660;<br>18.778	*MD=-26.686<br>0.095<br>-58.109;<br>4.737*	MD=0.021<br>0.998<br>-19.692; 19.735	MD=3.908<br>0.286<br>-3.314;<br>11.131	**MD=26.158<br>0.011<br>6.058;<br>46.259**	MD=0.641<br>0.483<br>-1.159; 2.441	MD=0.475<br>0.839<br>-4.143; 5.095	MD=-0.567<br>0.695<br>-3.429;<br>2.293	MD=0.032<br>0.426<br>-0.047;<br>0.112
**Diabetes**	**MD=-189.486<br>0.041<br>-371.087; -7.884**	MD=3.218<br>0.778<br>-19.429;<br>25.865	**MD=-32.606<br><0.001<br>-45.047; -<br>20.165**	MD=22.840<br>0.223<br>-14.111;<br>59.792	MD=11.833<br>0.411<br>-16.649; 40.316	MD=0.963<br>0.748<br>- 4.982;<br>6.910	MD=-<br>12.852<br>0.299<br>-37.343;<br>11.638	MD=0.038<br>0.968<br>-1.883; 1.960	MD=2.195<br>0.474<br>-3.882; 8.273	*MD=-2.633<br>0.057<br>-5.355;<br>0.088*	MD=0.097<br>0.171<br>-0.042;<br>0.236
**Cholesterol**	r=0.013<br>0.653<br>-0.043; 0.069	r=0.0117<br>0.686<br>-0.045; 0.068	r=-0.004<br>0.885<br>-0.061; 0.052	r=0.039<br>0.174<br>-0.0173;<br>0.096	r=0.032<br>0.268<br>-0.024; 0.088	r=0.009<br>0.761<br>-0.047;<br>0.065	r=0.010<br>0.721<br>-0.046;<br>0.067	r=0.0112<br>0.697<br>-0.045; 0.067	r=-0.002<br>0.949<br>-0.058; 0.054	r=0.014<br>0.624<br>-0.042;<br>0.070	r=-0.030<br>0.286<br>-0.087;<br>0.025
**Alcohol**	**r=0.327<br><0.001<br>0.275; 0.376**	r=0.009<br>0.766<br>-0.048; 0.065	r=0.005<br>0.861<br>-0.051; 0.061	r=0.031<br>0.284<br>-0.025; 0.087	r=-0.018<br>0.522<br>-0.075; 0.038	**r=0.062<br>0.030<br>0.006;<br>0.119**	**r=0.096<br><0.001<br>0.039; 0.152**	**r=0.086<br>0.003<br>0.030; 0.142**	r=0.043<br>0.132<br>-0.013; 0.100	r=0.027<br>0.337<br>-0.029; 0.08	r=-0.017<br>0.539<br>-0.074;<br>0.039
**Soft drinks**	**r=0.284<br><0.001<br>0.232; 0.337**	**r=0.093<br>0.001<br>0.036; 0.149**	**r=0.147<br><0.001<br>0.091; 0.202**	**r=0.069<br>0.017<br>0.012; 0.125**	r=0.045<br>0.115<br>-0.011; 0.102	r=0.045<br>0.123<br>-0.012;<br>0.101	**r=0.076<br>0.008<br>0.019; 0.132**	**r=0.100<br><0.001<br>0.043; 0.156**	**r=0.063<br>0.029<br>0.006; 0.119**	**r=0.089<br>0.002<br>0.033;<br>0.145**	r=0.035<br>0.217<br>-0.021;<br>0.092
**Energy intake**	-	**r=0.301<br><0.001<br>0.249; 0.352**	**r=0.586<br><0.001<br>0.548; 0.622**	**r=0.294<br><0.001<br>0.242; 0.345**	**r=0.426<br><0.001<br>0.378; 0.471**	**r=0.286<br><0.001<br>0.233;<br>0.337**	**r=0.688<br><0.001<br>0.657; 0.717**	**r=0.239<br><0.001<br>0.184; 0.291**	**r=0.245<br><0.001<br>0.190; 0.297**	**r=0.251<br><0.001<br>0.197;<br>0.304**	r=0.019<br>0.493<br>-0.036;<br>0.076
**Dairy products**	-	-	**r=0.127<br><0.001<br>0.070; 0.182**	**r=0.114<br><0.001<br>0.057; 0.171**	**r=0.134<br><0.001<br>0.078; 0.189**	r=0.037<br>0.190<br>-0.018;<br>0.09	**r=0.097<br><0.001<br>0.040; 0.153**	r=0.020<br>0.491<br>-0.036; 0.076	**r=0.066<br>0.021<br>0.010; 0.123**	**r=0.069<br>0.015<br>0.013;<br>0.126**	r=0.017<br>0.560<br>-0.039;<br>0.073

**Sugar foods**	-	-	-	**r=0.071<br>0.013<br>0.015; 0.128**	**r=0.073<br>0.011<br>0.016; 0.129**	r=0.035<br>0.226<br>-0.021;<br>0.091	**r=0.226<br><0.001<br>0.171; 0.279**	**r=0.114<br><0.001<br>0.057; 0.169**	**r=0.086<br>0.003<br>0.029; 0.142**	**r=0.109<br><0.001<br>0.052;<br>0.164**	r=0.040<br>0.167<br>-0.016;<br>0.096
**Fruits**	-	-	-	-	**r=0.225<br><0.001<br>0.170; 0.278**	**r=0.171<br><0.001<br>0.115;<br>0.225**	**r=0.122<br><0.001<br>0.065; 0.177**	r=-0.011<br>0.710<br>-0.067;<br>0.046	**r=0.093<br>0.001<br>0.037;<br>0.1499**	r=0.007<br>0.797<br>-0.049;<br>0.064	r=-0.004<br>0.874<br>-0.061;<br>0.052
**Vegetables**	-	-	-	-	-	**r=0.283<br><0.001<br>0.230;<br>0.334**	**r=0.203<br><0.001<br>0.148; 0.256**	**r=0.068<br>0.017<br>0.012; 0.125**	**r=0.278<br><0.001<br>0.225; 0.330**	**r=0.172<br><0.001<br>0.116;<br>0.226**	r=-0.001<br>0.946<br>-0.058;<br>0.054
**Legumes**	-	-	-	-	-	-	**r=0.198<br><0.001<br>0.143; 0.252**	**r=0.068<br>0.018<br>0.011; 0.124**	**r=0.149<br><0.001<br>0.093; 0.204**	**r=0.059<br>0.040<br>0.002;<br>0.115**	r=0.044<br>0.124<br>-0.012;<br>0.101
**Grains**	-	-	-	-	-	-	-	**r=0.114<br><0.001<br>0.058; 0.170**	**r=0.077<br>0.007<br>0.020; 0.133**	**r=0.091<br>0.002<br>0.035;<br>0.147**	r=0.025<br>0.373<br>-0.031;<br>0.082
**Fried foods**	-	-	-	-	-	-	-	-	r=-0.016<br>0.579<br>-0.072;<br>0.040	**r=0.109<br><0.001<br>0.053;<br>0.165**	r=-0.009<br>0.756<br>-0.065;<br>0.047
**Fish**	-	-	-	-	-	-	-	-	-	**r=0.145<br><0.001<br>0.089;<br>0.200**	r=-0.026<br>0.364<br>-0.082;<br>0.030
**Eggs**	-	-	-	-	-	-	-	-	-	-	r=-0.022<br>0.441

											-0.079; 0.034

Note. r: Pearson correlation coefficient; MD: Mean Difference; P: P-value; 95%CI: 95% Confidence Interval. In **bold** the significant results (p<0.05), in *italic* the suggestive ones (0.05<p<0.10).

Table S3: "grains" food group profiling by quartiles stratification on white meat intake

	White meat intake (g/die) (Overall, n=1192)					White meat intake (g/die) (Males, n=509)					White meat intake (g/die) (Females, n=683)				
**"Grains" foods**	**1st q [0-16.7]<br>(n=298)**	**2nd q (16.7-28.2]<br>(n=298)**	**3rd q (28.2- 44.6]<br>(n=299)**	**4th q (44.7-245]<br>(n=297)**	**P-value**	**1st q [0-17.4]<br>(n=128)**	**2nd q (17.4-29.7]<br>(n=127)**	**3rd q (29.7- 47.6]<br>(n=127)**	**4th q (47.6-245]<br>(n=127)**	**P-value**	**1st q [0-16.4]<br>(n=171)**	**2nd q (16.4-27]<br>(n=171)**	**3rd q (27.1- 43.2]<br>(n=170)**	**4th q (43.2-197.6]<br>(n=171)**	**P-value**
Grains (g/die)	171.50±97.524	178.842±94.251	176.86±81.99	207.34±112.365	**<0.001**	186.95±109.54	195.51±97.466	199.72±89.584	232.586±127.35	**0.004**	159.53±86.44	169.42±89.56	162.90±80.035	182.78±90.075	*0.431*
PASTASCIUTTA (g/die)	28.638±32.38	28.02±29.041	32.140±33.24	35.38±35.376	**0.021**	36.07±36.035	39.601±36.871	42.185±36.483	46.849±40.096	0.133	23.11±28.35	22.05±21.52	22.53±25.46	26.30±28.677	0.431
EGG PASTA (g/die)	2.677±5.954	2.100±5.307	2.877±7.078	2.677±6.847	0.475	2.842±6.303	2.559±8.099	3.551±7.015	4.362±9.035	0.243	2.69±5.792	2.108±5.66	1.832±4.088	1.479±4.298	0.15
FILLED PASTA (g/die)	8.850±11.591	8.492±11.097	8.722±11.582	9.283±12.577	0.869	8.230±11.902	7.937±13.855	7.344±9.903	11.309±13.860	*0.053*	9.04±10.86	9.369±10.57	9.142±11.383	8.185±11.453	0.771
RICE (g/die)	6.819±8.213	8.003±8.141	8.081±8.834	10.304±11.274	**<0.001**	6.815±9.021	8.650±9.901	8.162±9.825	11.935±13.246	**0.001**	6.78±7.497	7.787±7.129	7.882±7.741	8.995±9.290	*0.086*
RICE SALAD (g/die)	0.445±0.983	0.573±1.165	0.517±0.881	0.685±1.435	*0.070*	0.493±0.953	0.655±1.288	0.562±0.917	0.780±1.888	0.339	0.41±1.005	0.518±1.069	0.478±0.772	0.609±1.032	0.315
PASTA IN SOUP (g/die)	1.812±3.521	2.244±3.336	2.836±4.507	2.948±5.608	**0.004**	1.855±3.681	3.003±4.212	2.913±4.565	3.613±7.520	*0.058*	1.811±3.395	1.990±3.017	2.557±4.285	2.338±3.335	0.202
POLENTA (g/die)	0.446±1.059	0.405±1.144	0.425±1.206	0.523±1.827	0.723	0.417±0.809	0.503±1.351	0.316±0.633	0.538±1.643	0.445	0.466±1.211	0.397±1.115	0.471±1.411	0.484±1.940	0.947
PIZZA CUT (g/die)	1.254±3.359	1.120±4.115	1.608±6.475	1.060±2.714	0.427	1.802±4.137	1.320±5.424	1.825±7.793	0.911±2.420	0.464	0.953±2.876	0.822±2.415	1.454±5.199	1.209±3.185	0.371
PIZZA from PIZZERIA	12.220±16.605	14.364±14.572	17.702±18.219	21.733±25.041	**<0.001**	13.964±17.478	15.834±15.270	22.107±26.828	23.495±23.832	**<0.001**	10.699±15.82	14.032±14.305	15.579±16.566	18.725±20.554	**<0.001**
HOMEMADE PIZZA (g/die)	10.809±13.355	14.447±20.379	13.085±15.006	16.349±21.035	**0.002**	8.352±11.980	13.274±13.301	13.850±16.086	14.879±20.741	**0.005**	12.098±13.72	15.991±24.54	13.514±15.66	16.320±20.232	0.123
CHIPS OR SALADS (g/die)	1.011±2.159	1.353±2.848	1.917±3.923	2.559±4.175	**<0.001**	1.092±2.098	1.466±2.991	2.023±2.921	2.244±3.227	**0.005**	0.989±2.234	1.198±2.609	1.696±3.582	2.968±5.527	**<0.001**
COMMON BREADS (g/die)	21.703±45.423	21.139±38.709	19.70±30.721	21.122±35.066	0.929	26.975±57.341	25.09±45.905	22.989±33.675	27.214±42.745	0.873	18.108±33.789	17.35±31.562	18.919±30.684	15.413±24.621	0.741
WHITE BREAD (g/die)	30.424±48.475	29.460±52.820	23.35±40.749	30.554±52.678	0.221	38.149±52.788	31.65±51.947	31.498±47.221	37.327±60.347	0.633	24.872±44.089	29.46±54.05	16.565±36.603	24.27±42.845	*0.064*
WHOLE-GRAIN SANDWICHES (g/die)	11.740±23.012	11.783±26.288	11.14±22.452	12.804±31.069	0.889	9.299±17.293	8.558±19.527	8.062±16.459	12.934±36.639	0.357	13.394±26.275	13.33±29.65	13.78±26.79	13.38±26.053	0.999
WHOLE GRAIN CRACKERS (g/die)	0.963±3.752	1.537±4.779	1.304±3.471	2.202±6.213	**0.011**	1.027±2.820	1.668±5.654	0.971±2.881	2.410±7.661	*0.092*	0.967±4.332	1.192±3.676	1.739±4.152	2.053±4.881	*0.075*
WHOLE BREAD (g/die)	8.732±25.954	9.616±24.367	8.081±19.996	9.869±27.101	0.799	8.022±29.545	11.680±29.141	10.503±30.018	7.761±22.017	0.616	9.081±22.86	7.801±19.67	7.892±19.02	10.27±24.617	0.687
CONDITIONED BREADS	7.811±17.419	9.041±23.446	8.038±17.396	7.656±17.977	0.816	9.517±17.525	11.484±31.545	10.378±19.887	9.699±23.356	0.91	6.479±17.240	7.747±14.98	5.573±14.49	6.39±12.763	0.605
TOASTS (g/die)	6.477±11.366	6.054±9.646	6.093±10.458	6.884±10.633	0.75	5.614±9.941	5.094±9.491	4.467±9.800	5.854±10.578	0.693	6.923±12.248	6.563±9.58	7.741±11.06	7.62±10.536	0.715
CORN FLAKES (g/die)	2.861±8.891	3.206±9.391	2.117±6.917	3.591±9.683	0.208	2.167±7.565	2.136±7.884	1.294±5.839	3.058±9.909	0.371	3.497±9.846	3.348±9.42	3.444±8.95	3.79±9.207	0.974
MUESLI OATMEAL FRITTERS (g/die)	1.682±7.058	1.476±6.132	1.419±5.589	1.847±6.372	0.834	1.252±5.964	0.624±3.981	0.448±2.585	1.110±4.827	0.432	2.161±8.022	1.444±5.78	2.52±7.82	2.52±7.426	0.481
WHOLE GRAINS (g/die)	3.804±7.554	4.214±9.172	5.410±11.730	7.049±12.901	**<0.001**	2.846±6.749	2.627±5.338	4.091±11.533	4.172±8.716	0.316	4.518±8.184	4.730±9.22	7.183±13.52	9.067±14.648	**<0.001**
SEEDS (g/die)	0.321±1.062	0.175±0.677	0.279±0.764	0.254±0.856	0.2	0.140±0.594	0.088±0.482	0.171±0.557	0.124±0.639	0.703	0.458±1.293	0.169±0.59	0.399±0.943	0.381±1.037	**0.042**
**Outcome**															
MASLD (yes/no)	158/140	143/155	151/148	135/162	0.285	85/43	72/55	64/63	57/70	**0.004**	75/96	74/97	81/89	79/92	0.835

	(53%/47%)	(48%/52%)	(50.5%/49.5%)	(45.5%/54.5%)		(66.4%/33.6%)	(56.7%/43.3%)	(50.4%/49.6%)	(44.9%/55.1%)		(43.9%/56.1%)	(43.3%/56.7%)	(47.6%/52.4%)	(46.2%/53.8%)	

Note. Descriptive statistics are shown as mean±standard deviation or frequencies, as appropriate. q: quartile; n: sample size. In **bold** significant results ($p<0.05$), in *italics* the suggestive ones ($0.05<p<0.10$). MASLD: Metabolic dysfunction-associated steatotic liver disease.

Appendix: **R/dagitty code on the Direct Acyclic Graph (DAG) for theory driven minimal sufficient adjustment set of the confounders of the direct effect of the white meat intake on MASLD.**

```
dag {
"Dairy products" [adjusted,pos="-2.101,-0.269"]
"Fresh fruits" [adjusted,pos="0.062,1.458"]
"Fried foods" [adjusted,pos="-2.132,-1.270"]
"Kcal intake" [adjusted,pos="0.327,1.163"]
"Processed meat" [adjusted,pos="0.635,-0.368"]
"Red meat" [adjusted,pos="0.528,0.291"]
"Soft drinks" [adjusted,pos="-2.106,0.159"]
"Sugar foods" [adjusted,pos="0.135,-1.882"]
"White meat" [exposure,pos="0.546,-0.869"]
Age [adjusted,pos="-2.264,0.739"]
Alcool [adjusted,pos="-1.607,-1.765"]
BMI [adjusted,pos="0.582,1.076"]
Cholesterol [adjusted,pos="-1.522,1.324"]
Diabetes [adjusted,pos="-1.915,1.280"]
Education [adjusted,pos="-1.719,-1.284"]
Eggs [adjusted,pos="-1.239,-1.962"]
Fish [adjusted,pos="-0.288,-1.937"]
Grains [adjusted,pos="-2.041,-0.836"]
Legumes [adjusted,pos="0.558,-1.696"]
Margarin [adjusted,pos="-1.075,1.227"]
Sex [adjusted,pos="-0.330,-1.713"]
Smoke [adjusted,pos="-0.824,-1.706"]
MASLD [outcome,pos="-0.400,-0.315"]
Vegetables [adjusted,pos="-0.173,0.779"]
"Dairy products" -> MASLD
"Dairy products" <-> "Fresh fruits"
"Dairy products" <-> "Red meat"
"Dairy products" <-> "Sugar foods"
"Dairy products" <-> "White meat"
"Dairy products" <-> Eggs
"Dairy products" <-> Fish
"Dairy products" <-> Legumes
"Dairy products" <-> Vegetables
"Fresh fruits" <-> "Processed meat"
"Fresh fruits" <-> "Red meat"
"Fresh fruits" <-> Eggs
"Fresh fruits" <-> Fish
"Fresh fruits" <-> Grains
"Fresh fruits" <-> Legumes
"Fresh fruits" <-> Vegetables
"Fried foods" <-> "Red meat"
"Fried foods" <-> "White meat"
"Fried foods" <-> Eggs
"Fried foods" <-> Fish
"Fried foods" <-> Margarin
"Kcal intake" -> MASLD
"Kcal intake" <-> "Processed meat"
"Kcal intake" <-> "Red meat"
"Kcal intake" <-> "White meat"
"Processed meat" -> MASLD
"Processed meat" <-> "Sugar foods"
"Processed meat" <-> Cholesterol
"Processed meat" <-> Vegetables
"Red meat" -> "Processed meat"
"Red meat" -> MASLD
"Red meat" <-> "Sugar foods"
"Red meat" <-> Cholesterol
"Red meat" <-> Eggs
```

```
"Red meat" <-> Fish
"Red meat" <-> Grains
"Red meat" <-> Legumes
"Red meat" <-> Margarin
"Red meat" <-> Vegetables
"Soft drinks" -> MASLD
"Soft drinks" <-> "Sugar foods"
"Soft drinks" <-> Age
"Soft drinks" <-> Diabetes
"Sugar foods" -> MASLD
"Sugar foods" <-> "White meat"
"Sugar foods" <-> Diabetes
"Sugar foods" <-> Eggs
"Sugar foods" <-> Fish
"Sugar foods" <-> Grains
"White meat" -> "Processed meat"
"White meat" -> MASLD
"White meat" <-> Eggs
"White meat" <-> Fish
"White meat" <-> Grains
"White meat" <-> Legumes
Age -> MASLD
Alcool -> MASLD
Alcool <-> Education
Alcool <-> Smoke
BMI -> MASLD
Diabetes -> MASLD
Education -> MASLD
Education <-> Sex
Eggs <-> Fish
Eggs <-> Grains
Eggs <-> Legumes
Eggs <-> Vegetables
Fish <-> Grains
Fish <-> Legumes
Fish <-> Vegetables
Grains -> MASLD
Grains <-> Margarin
Grains <-> Vegetables
Legumes <-> Margarin
Legumes <-> Vegetables
Margarin -> MASLD
Margarin <-> Vegetables
Sex -> MASLD
Smoke -> MASLD
}
```

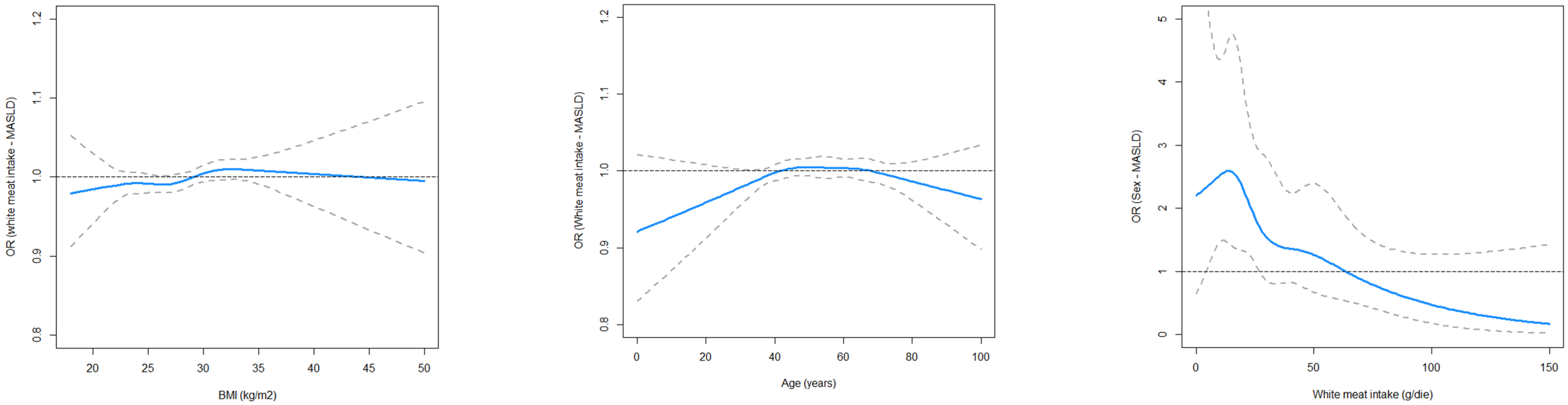

Figure S1. Interaction effects of sex, age and BMI with white meat intake on MASLD

In cyan is shown the dose response effect, whereas the 95% confidence bounds are presented in dashed gray. ORs were significant by whether their 95% confidence bounds did not involve the value “1”.

OR: odds ratio. MASLD: Metabolic dysfunction-associated steatotic liver disease.

The models are adjusted for the potential confounders included in the DAG-related minimal sufficient adjustment set.

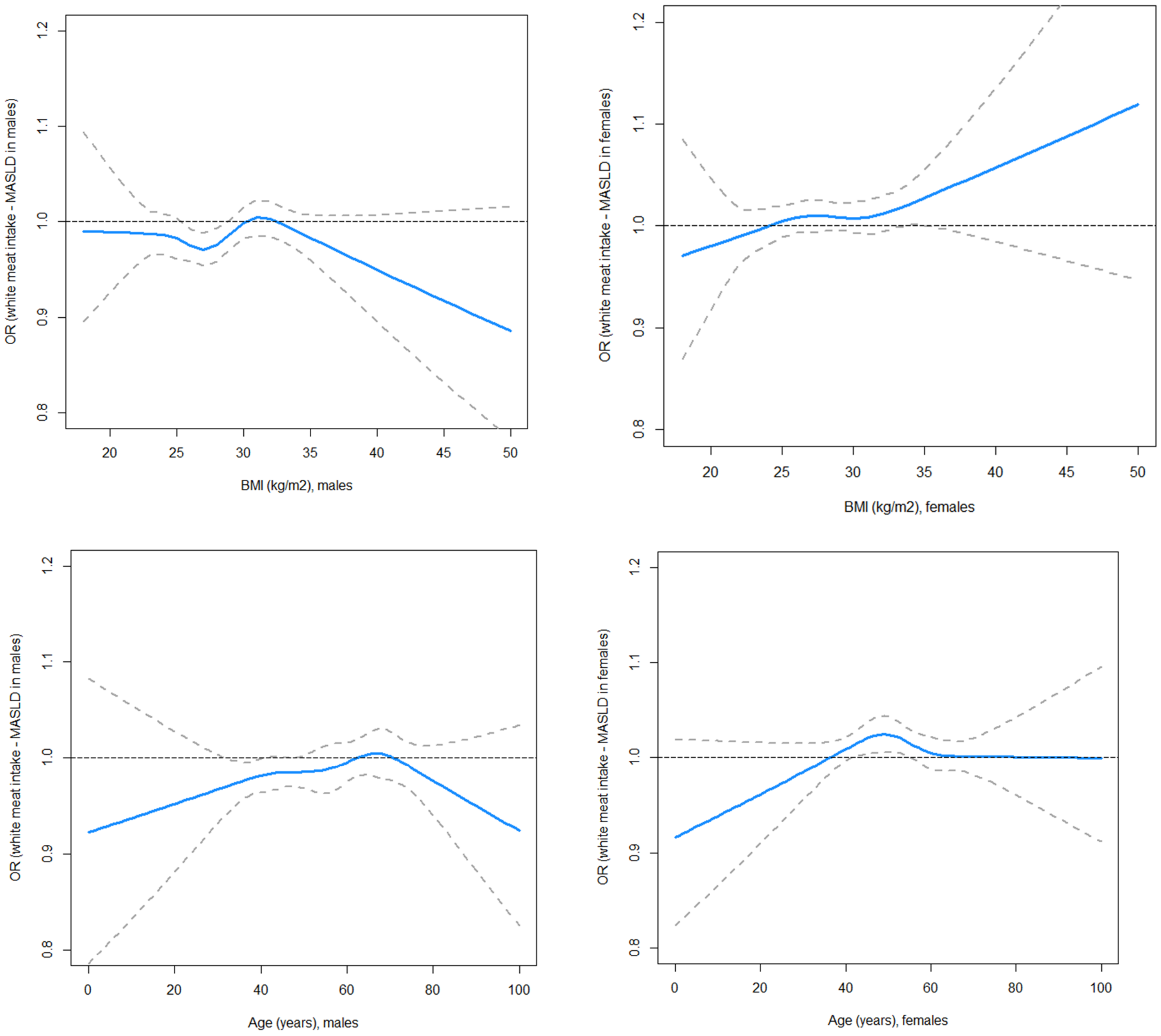
OR (white meat intake - MASLD in males)
BMI (kg/m2), males
OR (white meat intake - MASLD in females)
BMI (kg/m2), females
OR (white meat intake - MASLD in males)
Age (years), males
OR (white meat intake - MASLD in females)
Age (years), females

Figure S2. Interaction effects of age and BMI with white meat intake on MASLD by sex

In cyan is shown the dose response effect, whereas the 95% confidence bounds are presented in dashed gray. ORs were significant by whether its 95% confidence bounds did not involve the value "1": OR: odds ratio. MASLD: Metabolic dysfunction-associated steatotic liver disease.

The models are adjusted for the potential confounders included in the DAG-related minimal sufficient adjustment set.

nutrients

MDPI

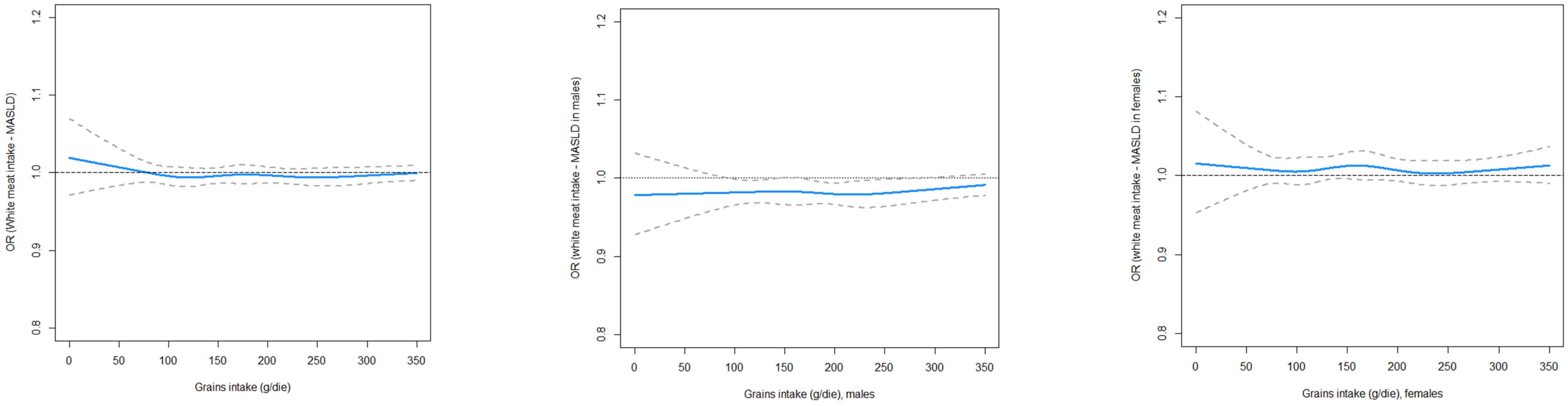

Figure S3. Interaction effects of "grains" food group with white meat intake on MASLD, overall and by sex

In cyan is shown the dose response effect, whereas the 95% confidence bounds are presented in dashed gray. ORs were significant by whether its 95% confidence bounds did not involve the value "1": OR: odds ratio. MASLD: Metabolic dysfunction-associated steatotic liver disease.

The models are adjusted for the potential confounders included in the DAG-related minimal sufficient adjustment set.
